# Dominant and Recessive Major *R* Genes Lead to Different Types of Host Cell Death During Resistance to *Xanthomonas oryzae* in Rice

**DOI:** 10.3389/fpls.2018.01711

**Published:** 2018-11-21

**Authors:** Jianbo Cao, Meng Zhang, Jinghua Xiao, Xianghua Li, Meng Yuan, Shiping Wang

**Affiliations:** ^1^National Key Laboratory of Crop Genetic Improvement, National Center of Plant Gene Research (Wuhan), Huazhong Agricultural University, Wuhan, China; ^2^Public Laboratory of Electron Microscopy, Huazhong Agricultural University, Wuhan, China

**Keywords:** major disease resistance gene, bacteria blight, autophagy-like cell death, vacuolar-mediated cell death, ultrastructure

## Abstract

The bacterial blight caused by *Xanthomonas oryzae* pv. *oryzae* (*Xoo*) is the most devastating bacterial disease of rice worldwide. A number of dominant major disease resistance (*MR*) genes and recessive *MR* genes against *Xoo* have been cloned and molecularly characterized in the last two decades. However, how these *MR* genes mediated-resistances occur at the cytological level is largely unknown. Here, by ultrastructural examination of xylem parenchyma cells, we show that resistances to *Xoo* conferred by dominant *MR* genes and recessive *MR* genes resulted in different types of programmed cell death (PCD). Three dominant *MR* genes *Xa1, Xa4*, and *Xa21* and two recessive *MR* genes *xa5* and *xa13* that encode very different proteins were used in this study. We observed that *Xa1*-, *Xa4*-, and *Xa21*-mediated resistances to *Xoo* were associated mainly with autophagy-like cell death featured by the formation of autophagosome-like bodies in the xylem parenchyma cells. In contrast, the *xa5*- and *xa13*-mediated resistances to *Xoo* were associated mainly with vacuolar-mediated cell death characterized by tonoplast disruption of the xylem parenchyma cells. Application of autophagy inhibitor 3-methyladenine partially compromised *Xa1*-, *Xa4*-, and *Xa21*-mediated resistances, as did Na_2_HPO_4_ alkaline solution to *xa5*- and *xa13*-mediated resistances. These results suggest that autophagy-like cell death is a feature of the dominant *MR* gene-mediated resistance to *Xoo* and vacuolar-mediated cell death is a characteristic of the recessive *MR* gene-mediated resistance.

## Introduction

Plant resistance against pathogens can be genetically classified into two classes based on the strength of resistance: qualitative or complete resistance conferred by major disease resistance (*MR*) genes and quantitative or partial resistance mediated by multiple genes or quantitative trait loci (Kou and Wang, 2010; Zhang and Wang, 2013). The molecular mechanisms of plant disease resistance are explained, in general, with a two-tiered innate immune system: pathogen-associated molecular pattern-triggered immunity (PTI) or plant-derived damage-associated molecular PTI or basal resistance, and effector-triggered immunity (ETI) or gene-for-gene resistance (Jones and Dangl, 2006; Thomma et al., 2011; Monaghan and Zipfel, 2012). PTI is initiated by plasma membrane-localized plant pattern recognition receptors (PRRs), which are receptor-kinase proteins or receptor-like proteins, and ETI is initiated by cytoplasmic nucleotide-binding (NB)–leucine-rich repeat (LRR)-type resistance proteins (Jones and Dangl, 2006; Macho and Zipfel, 2014). Thus, in general, PTI is quantitative resistance and ETI is qualitative resistance in many plant–pathogen pathosystems (Zhang and Wang, 2013). However, rice and biotrophic *Xanthomonas oryzae* pv. *oryzae* (*Xoo*), which causes the most devastating bacterial disease of rice worldwide, are a unique pathosystem for rice qualitative resistance against *Xoo* (Zhang and Wang, 2013). The rice *MR* genes resistant to *Xoo* can be an ETI or a PTI or other mechanisms that cannot be explained by ETI and PTI (Zhang and Wang, 2013; Ke et al., 2017).

Except for the rice-*Xoo* pathosystem, earlier studies have shown that plant qualitative resistance to biotrophic pathogens frequently featured a rapid hypersensitive response (HR), which is characterized by rapid and localized cell death to restrict pathogen replication during the early stage of the plant–pathogen interaction (Pontier et al., 1998; Mur et al., 2008). Further studies have revealed that HR is often, but not always, a part of ETI initiated by NB-LRR proteins (Coll et al., 2011). HR-associated cell death is a kind of programmed cell death (PCD). Evolutionarily conserved autophagy, which is intracellular self-digestion of cytoplasmic components characterized by the formation of membrane-bound autophagosomes carrying a portion of the cytoplasm to be degraded or organelle permeabilization, has been observed to be involved in plant PCD (Dickman and Fluhr, 2013; Kabbage et al., 2017). The autophagosomes have different ultrastructures: (1) the vacuolar membrane (tonoplast)-bound body (microautophagy) formed by a portion of the cytoplasm, cytoplasmic vesicles or organelles in the vacuole; (2) the double-membrane body (macroautophagy) formed by a portion of cytoplasm bound by an endoplasmic reticulum-like tubule derived double-membrane in the cytoplasm; (3) the multilamellar body formed by many membranes bound by a single membrane in the cytoplasm (van Doorn and Papini, 2013). In addition, vacuolar-mediated PCD, in which the tonoplast integrity is compromised or the tonoplast is fused with the plasma membrane resulting in the release of vacuolar components into the cytoplasm or the extracellular space leading to cell death, also occurs in HR (Hara-Nishimura and Hatsugai, 2011; Dickman and Fluhr, 2013). In contrast, plant necrosis is characterized by shrinkage of the protoplast and rupture of the plasma membrane (van Doorn et al., 2011).

One of the features that makes the qualitative resistance of rice to *Xoo* unique from other pathosystems is that one third of the 41 *MR* genes identified thus far are genetically recessive (Zhang and Wang, 2013; Ke et al., 2017). Eleven (*Xa1, Xa3/Xa26, Xa4, xa5, Xa10, xa13, Xa21, Xa23, xa25, Xa27*, and *xa41*) of these 41 genes, which have been cloned and molecularly characterized at present, are shown to encode diverse types of proteins. Among the dominant genes, *Xa1* encodes a classic NB-LRR-type protein (Yoshimura et al., 1998), *Xa3/Xa26* and *Xa21* encode plasma membrane-localized LRR receptor kinase-type proteins (Song et al., 1995; Sun et al., 2004), *Xa4* encodes a cell wall-associated protein kinase (Hu et al., 2017). The recessive gene *xa5* encodes a mutated basal transcriptional factor IIA gamma (TFIIAγ) subunit 5 (TFIIAγ5^V39E^) (Iyer and McCouch, 2004; Yuan et al., 2016), and *xa13, xa25*, and *xa41* encode MtN3/saliva/SWEET-type membrane proteins with XA13 and XA25 localized in the plasma membrane (Chu et al., 2006; Liu et al., 2011; Hutin et al., 2015; Cheng et al., 2017). The dominant *MR* genes *Xa1, Xa4, Xa21* and the recessive *MR* genes *xa5, xa13* are race-specifically resistant to *Xoo*, while the recessive genes *xa1, xa4, xa21* and the dominant *Xa5, Xa13* are susceptible to *Xoo* (Zhang and Wang, 2013).

*Xa27, xa13*, and *xa5* can trigger a HR (although the HR of *xa5* is weak) leading to the brown symptoms on infected rice leaves after infiltrating inoculation with avirulent *Xoo* strains (Gu et al., 2005; Yang et al., 2006; Iyer-Pascuzzi et al., 2008). The ectopically expressed *Xa10* and *Xa23* can only induce HR in *Nicotiana benthamiana* (Tian et al., 2014; Wang et al., 2015). Nonetheless, the formation of HR in rice xylem vessel tissue against *Xoo* bacteria, which multiply in xylem vessels of rice leaves under natural infection conditions (Kou and Wang, 2010), needs to be investigated.

To address the HR of rice *MR* gene-mediated resistance to *Xoo*, we compared tissue phenotypes and ultrastructural morphologies of rice leaves from lines containing the dominant *Xa1, Xa4*, and *Xa21 MR* genes and the recessive *xa5* and *xa13 MR* genes. We found that autophagy-like cell death is the major characteristic in dominant *MR* gene-mediated resistance and that vacuolar-mediated cell death is the main feature in recessive *MR* gene-mediated resistance. These findings suggest that the different types of HR-PCD contribute to the resistance of rice against *Xoo* by different *MR* genes.

## Materials and Methods

### Rice Materials

IRBB1, IRBB4, IRBB21, IRBB5, and IRBB13 are near-isogenic rice lines (NILs) carrying dominant *MR* genes *Xa1, Xa4*, and *Xa21* and recessive *MR* genes *xa5* and *xa13*, respectively, in the genetic background of the rice variety IR24. Each of these five lines confers race-specific resistance to *Xoo* bacteria with different resistance spectra (Zhang and Wang, 2013; Hu et al., 2017).

### Pathogen Inoculation

Rice plants were inoculated with 10^9^ cells ml^-1^ of *Xoo* strains T7174 (Japanese race (1), PXO61, PXO86, PXO112, PXO99, or PXO341 (Philippine race 1, 2, 5, 6 or 10) suspension at either the 4-leaf stage (rice lines IRBB4, IRBB5, IRBB13, and IR24) or 7-leaf stage (rice lines IRBB1, IRBB21, and IR24) by the leaf-clipping method (Chen et al., 2002). For mock treatment, water without *Xoo* was used by clipping rice leaves. Disease was scored by measuring the lesion length at 2 weeks after inoculation. To study the cell responses to *Xoo* and the effects of 3-methyladenine (3-MA) and Na_2_HPO_4_ on resistance, rice plants were inoculated by infiltrating leaves with a bacterial suspension of *Xoo* using a needleless syringe method (Schaad et al., 1996). The bacterial suspension with 10^9^ cells ml^-1^ contained 5 mM 3-MA (Sigma, SIGMA-ALDRICH, St. Louis, MO, United States) or 2 mM Na_2_HPO_4_ (Sinopharm, Signopharm Chemical Reagent Co., Ltd., Shanghai, China). For mock treatment, leaves were infiltrated only by 5 mM 3-MA or 2 mM Na_2_HPO_4_ solution. Disease was scored by counting the number of infiltrating sites with water-soaked symptom at 3 days after inoculation. The inoculated leaves were photographed using scientific scanner (Image Scanner III, GE, Sweden). All the inoculation of plants with *Xoo* was biologically repeated at least twice with similar results, and one replicate was shown.

### Transmission Electron Microscopy

The ultrastructure of rice leaf cells was studied by transmission electron microscopy. The leaves were sampled at 0 day after inoculation (sampling at 1 h after inoculation) (0 DAI), 3 DAI, 5 DAI, and 14 DAI. The leaf tissues at the inoculation sites were cut into 1 × 3 mm pieces and fixed in 2.5% (w/v) glutaraldehyde in 0.1 M phosphate buffer solution (PBS) (pH 7.2) at 4°C overnight. The fixed tissues were washed in PBS three times for 30 min each at room temperature (20–25°C), postfixed for 2 h in 1% osmium tetroxide, dehydrated in a graded series of acetone, infiltrated with Spurr resin (SPI, SPI Chem, West Chester, PA, United States), and polymerized at 65°C for 48 h. The samples were cut into ultrathin sections (60–70 nm thick), stained with 2% uranyl acetate, and examined with a Hitachi transmission electron microscope (H-7650; Hitachi, Japan) at 80 kv. Each sample had 3 biological replicates with each replicate having at least 3 ultrathin sections observed under the electron microscope. To quantify the cells containing autophagosome-like body, tonoplast disruption and protoplast shrinkage, 208–520 xylem parenchyma cells were observed from at least six or nine leaf xylem veins of six or nine plants in two or three independent inoculations. In each xylem vein, the total xylem parenchyma cells of one vein were observed, then the frequencies (%) of the cells with the above three structures in the total xylem parenchyma cells were calculated. 57–80 mesophyll cells (approximately 10 cells from each plant) were observed and calculated for the frequencies (%) of cells with the above three structures from six leaves of six plants in two independent inoculations.

### Expression Analysis of Autophagy-Related Genes and Vacuolar Processing Enzyme Gene

The 3-cm leaf fragments next to the inoculation sites were used for RNA isolation. Quantitative reverse transcription-PCR (qRT-PCR) was conducted as described previously using gene-specific primers (Supplementary Table S1; Qiu et al., 2007). The expression level of actin gene was used to standardize the RNA sample for each qRT-PCR, and then the expression level relative to the control was calculated. The assays were biologically repeated twice with similar results, and only one replicate was presented.

### Statistical Analysis

The significant differences of lesion length, gene expression level and the number of cells with autophagosome-like body, tonoplast disruption or protoplast shrinkage ultrastructures between resistant and susceptible plants, were assessed using pairwise Student’s *t*-test in Excel (Microsoft^1^).

## Results

### The Leaf Tissue Morphology at Rice–*Xoo* Interaction Sites

**FIGURE 1:**
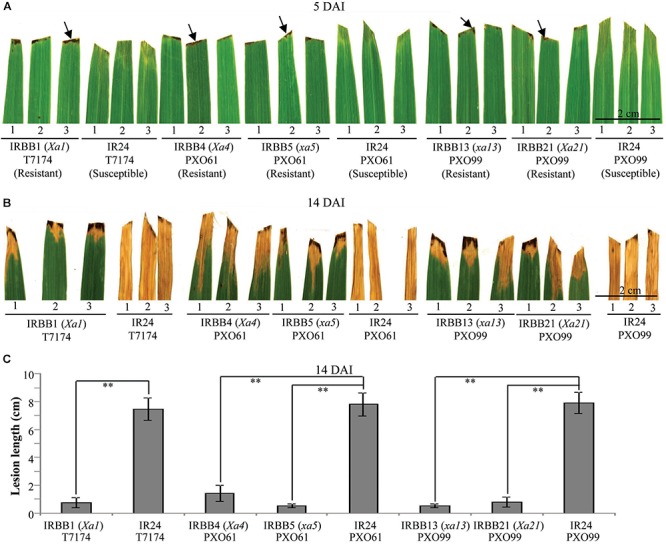
Phenotypes of different rice line–*Xoo* strain interactions. DAI, day after infection; arrow, hypersensitive response-like lesion. **(A,B)** Leaf responses of resistant rice lines IRBB1, IRBB4, IRBB5, IRBB13, and IRBB21 and susceptible rice line IR24 inoculated with *Xoo* strain T7174, PXO61, or PXO99 at 5 and 14 DAI. **(C)** Lesion length of different rice lines infected with *Xoo* at 14 DAI. Bars represent mean (12 to 16 leaves from four plants) ± standard deviation (SD). The double asterisk (^∗∗^) stands for the significant difference between resistant and susceptible plants at *P* < 0.01.

Leaf tissue is a major infection site of *Xoo* (Niño-Liu et al., 2006). To study whether different types of rice *MR* gene-mediated resistance against *Xoo* have different types of cell death, we first analyzed the leaf morphology of rice–*Xoo* interaction sites in resistant IRBB1, IRBB4, IRBB5, IRBB13 and IRBB21 lines each carrying a different *MR* gene and susceptible line IR24 against *Xoo* strains T7174, PXO61, or PXO99 (Figure 1). At 5 days after infection (DAI), the brown HR-like lesions appeared on all the inoculation sites of rice leaves of the NILs carrying *MR* genes, while the inoculation sites of susceptible IR24 leaves formed approximately 0.5-cm-long chlorotic water-soaked symptoms (Figure 1A). At 14 DAI, all the infection sites of leaf tissue turned into yellow lesions in the resistant rice lines and the susceptible rice line (Figure 1B). The average lesion length of IR24 was about 6-fold longer than that of IRBB1, IRBB4, IRBB5, IRBB13, and IRBB21 at 14 DAI (Figure 1C).

### Ultrastructural Morphotypes of Xylem Parenchyma Cells in Different *MR* Gene-Mediated Resistances to *Xoo*

**FIGURE 2:**
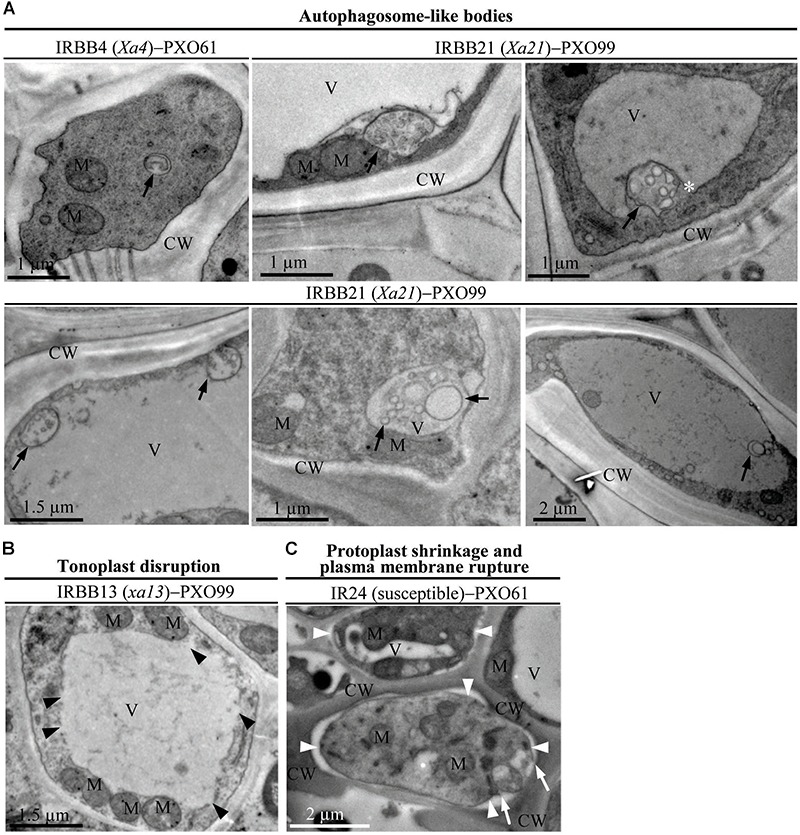
The abnormal ultrastructures in rice xylem parenchyma cells after *Xoo* infection at 3 DAI. XV, xylem vessel; V, vacuole; CW, cell wall; M, mitochondrion. **(A)** Autophagosome-like bodies (dark arrows showing double-membrane-like body or single-membrane-like body) in the cytoplasm and vacuoles during resistant reactions. **(B)** Tonoplast disruption (dark arrowheads) in the resistant reaction. **(C)** Protoplast shrinkage (white arrowheads) and plasma membrane rupture (white arrows) in the susceptible reaction.

*Xoo* multiply and spread in the xylem vessels of rice leaves (Kou and Wang, 2010). The leaf xylem parenchyma cells surrounding xylem vessels are in direct contact with *Xoo* (Niño-Liu et al., 2006). Thus, we examined the ultrastructure of xylem parenchyma cells in the process of cell death after *Xoo* infection in NILs by transmission electron microscopy. We observed three types of abnormal ultrastructures in xylem parenchyma cells of infection sites at 3 DAI (Figures 2A–C). The first type of abnormal ultrastructure was observed mostly in resistant IRBB4 and IRBB21 lines, which showed autophagosome-like bodies formed by autophagy processes including double-membrane-like bodies in the cytoplasm, single-membrane-bound bodies containing multiple small vesicles in the cytoplasm, and tonoplast-bound bodies in vacuoles (Figure 2A). The second type of abnormal ultrastructure was tonoplast disruption that was commonly observed in the resistant IRBB13 line (Figure 2B). The third type of abnormal structure was protoplast shrinkage and rupture of the plasma membrane observed in the susceptible IR24 line (Figure 2C).

**FIGURE 3:**
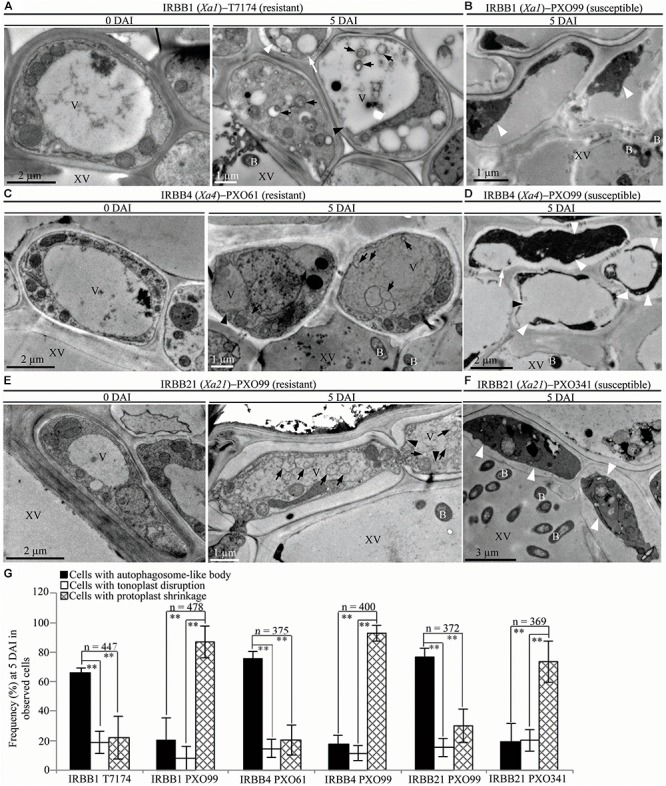
Autophagosome-like body ultrastructural feature of xylem parenchyma cells in dominant *MR* genes *Xa1, Xa4*, or *Xa21* mediated-resistance. B, *Xoo* bacterium; V, vacuole; XV, xylem vessel; dark arrow, autophagosome-like body; dark arrowhead, tonoplast disruption; white arrowhead, protoplast shrinkage; white arrow, rupture of plasma membrane. **(A–F)** Many autophagosome-like bodies in xylem parenchyma cells of IRBB1, IRBB4, and IRBB21 plants at 5 days after inoculation (DAI) with *Xoo* strains T7174, PXO61, or PXO99 comparison with plants at 0 DAI and plants susceptible reaction to compatible strains PXO99, PXO341. **(G)** The percentage of cells with autophagosome-like bodies, tonoplast disruption, and protoplast shrinkage in micrographs of xylem parenchyma cell in rice leaves at 5 DAI. Data represent mean (at least nine leaf xylem parenchyma cells were observed from nine different plants in two or three independent inoculations) ± SD. The double asterisk (^∗∗^) stands for a significant difference between frequency of cells with autophagosome-like body and frequency of cells with tonoplast disruption or protoplast shrinkage at *P* < 0.01. *n*, the total number of observed cells.

**FIGURE 4:**
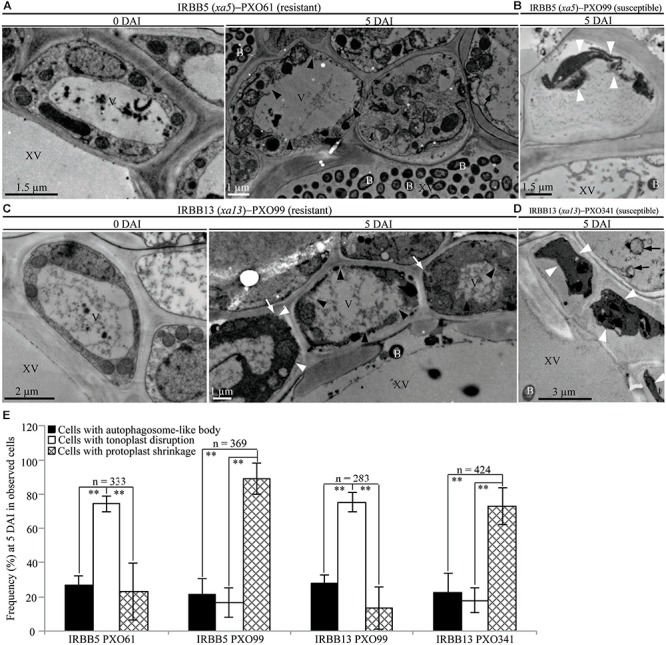
Tonoplast disruption ultrastructure of xylem parenchyma cells in recessive *MR* genes *xa5* and *xa13* mediated-resistance. V, vacuole; XV, xylem vessel; and B, *Xoo* bacterium. Dark arrow, autophagosome-like body; dark arrowhead, tonoplast disruption; white arrowhead, protoplast shrinkage; and white arrow, rupture of plasma membrane. **(A–D)** Many xylem parenchyma cells with tonoplast disruption in IRBB5 and IRBB13 plants at 5 days after inoculation (DAI) with *Xoo* strains PXO61 and PXO99 comparison with plants at 0 DAI and plants susceptible reaction to compatible strains PXO99, PXO341. **(E)** The percentage of cells with autophagosome-like bodies, tonoplast disruption and protoplast shrinkage in micrographs of xylem parenchyma cell in rice leaves at 5 DAI. Data represents mean (at least nine leaf xylem parenchyma cells were observed from nine different plants in two or three independent inoculations) ± SD. The double asterisk (^∗∗^) stands for a significant difference between frequency of cells with tonoplast disruption and frequency of cells with autophagosome-like body or protoplast shrinkage at *P* < 0.01. *n*, the total number of observed cells.

**FIGURE 5:**
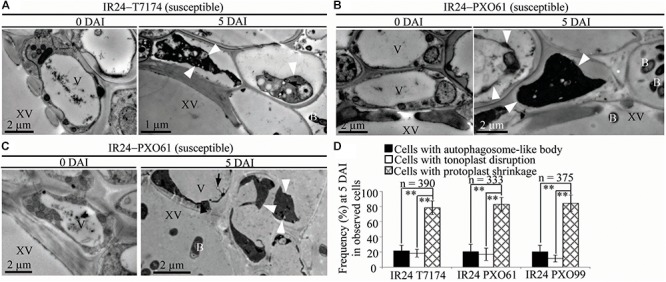
Necrosis ultrastructural features of xylem parenchyma cells in susceptible reactions after *Xoo* infection. V, vacuole; XV, xylem vessel; and B, *Xoo* bacterium. Dark arrow, autophagosome-like body; dark arrowhead, tonoplast disruption; white arrowhead, protoplast shrinkage; and white arrow, rupture of plasma membrane. **(A–C)** Many xylem parenchyma cells with protoplast shrinkage in IR24 plants at 5 days after inoculation (DAI) susceptible to *Xoo* strains T7174, PXO61, PXO99 comparison with plants at 0 DAI. **(D)** The percentage of cells with autophagosome-like bodies, tonoplast disruption, and protoplast shrinkage in micrographs of xylem parenchyma cells in rice leaves at 5 DAI. Data represent mean (at least nine leaf xylem parenchyma cells were observed from nine different plants in two or three independent inoculations) ± SD. The double asterisk (^∗∗^) stands for a significant difference between frequency of cells with protoplast shrinkage and frequency of cells with tonoplast disruption or autophagosome-like body at *P* < 0.01. *n*, the total number of observed cells.

To study whether the dominant or recessive *MR* gene-mediated resistance and the susceptible reaction are associated with different abnormal structures in rice-*Xoo* interactions, we counted the xylem parenchyma cells containing the three abnormal ultrastructures in all the rice lines at 0 and 5 DAI. There were integrated protoplasts and intact organelles in xylem parenchyma cells of all the rice lines and no significant difference in the numbers of xylem parenchyma cells with the three abnormal ultrastructures at 0 DAI among different lines (Figures 3A,C,E, 4A,C, 5A–C and Supplementary Figures S1, S2). However, at 5 DAI, autophagosome-like bodies, tonoplast disruption, and protoplast shrinkage were observed in xylem parenchyma cells of all the rice lines, but the frequencies of cells with the three ultrastructures were very different among these rice–*Xoo* interactions (Figures 3–5 and Supplementary Figure S2). In IRBB1, IRBB4, and IRBB21 lines resistant to *Xoo* strains T7174, PXO61 or PXO112, PXO99, or PXO61, autophagosome-like bodies were the major feature in xylem parenchyma cells (Figures 3A,C,E and Supplementary Figures S2A–B). The number of rice cells containing autophagosome-like bodies was 4- and 3-fold higher than the number of cells containing tonoplast disruption and plasmolysis, respectively, in IRBB1; was 5- and 4-fold higher than the number of cells containing tonoplast disruption and plasmolysis, respectively, in IRBB4; and was 5- and 3-fold higher than the number of cells containing tonoplast disruption and plasmolysis, respectively, in IRBB21 (Figure 3G and Supplementary Figure S2D). However, protoplast shrinkage was the major features in xylem parenchyma cells in IRBB1, IRBB4 and IRBB21 lines susceptible to compatible *Xoo* strain PXO99, PXO341 (Figures 3B,D,F). The number of cells with protoplast shrinkage was 4- and 6-fold higher than the number of cells containing autophagosome-like bodies and tonoplast disruption (Figure 3G).

In contrast, tonoplast disruption was observed to be the major feature in xylem parenchyma cells during the IRBB5, IRBB13 lines resistant to *Xoo* strains PXO61 or PXO86, PXO99 (Figures 4A,C and Supplementary Figure S2C). The number of rice cells containing tonoplast disruption was 3-fold higher than the number of cells containing autophagosome-like bodies or cells showing protoplast shrinkage in IRBB5, and was 3-fold higher than the number of cells containing autophagosome-like bodies and 6-fold higher than cells showing protoplast shrinkage in IRBB13 (Figure 4E and Supplementary Figure S2D). However, protoplast shrinkage was also the major features in xylem parenchyma cells in IRBB5, IRBB13 lines susceptible to compatible *Xoo* strains PXO99, PXO341 (Figures 4B,D). The number of cells with protoplast shrinkage was 4- and 6-fold higher than the number of cells containing autophagosome-like bodies and tonoplast disruption (Figure 4E). Meanwhile, the average lesion length of rice lines containing *MR* genes inoculated with compatible strains all exceeded 6 cm at 14 DAI (Supplementary Figure S3).

Furthermore, protoplast shrinkage was observed to be the major ultrastructure feature in xylem parenchyma cells in rice susceptible reactions to *Xoo* (Figure 5). Many xylem parenchyma cells showed protoplast shrinkage in IR24 that was susceptible to *Xoo* strains T7174, PXO61, and PXO99 (Figures 5A–C). The number of cells with protoplast shrinkage was 4- and 7-fold higher than the number of cells containing autophagosome-like bodies and tonoplast disruption, respectively, in all IR24–*Xoo* interactions (Figure 5D).

### Expression of Autophagy-Related Genes and Vacuolar Processing Enzyme Gene in Different Resistances to *Xoo*

Autophagy-related genes (*ATG*) control the formation of autophagosome-like body in plants (Liu et al., 2005; Xia et al., 2011). Vacuolar processing enzyme genes (*VPE*) regulate vacuolar mediated cell death in plant-pathogens interactions and H_2_O_2_-induced PCD (Hatsugai et al., 2004; Deng et al., 2011). We analyzed transcription level of autophagy-related genes *OsATG5* and *OsATG7, VPE* gene *OsVPE2* in IRBB1, IRBB4, IRBB21, and IRBB13 lines resistant to *Xoo* strains T7174, PXO61, and PXO99 (Supplementary Figure S4). On 8 and 24 h after inoculation, *OsATG5* and *OsATG7* were markedly induced to higher levels in IRBB1, IRBB4, and IRBB21 lines than that in susceptible IR24 control lines; *OsVPE2* was not induced in IRBB1, IRBB21 lines but was markedly induced to higher levels in IRBB13 plants compared to IR24 (Supplementary Figure S4).

### Ultrastructural Morphotypes of Xylem Parenchyma Cells at Late Stage of Rice–*Xoo* Interaction and at Mock Treatment

**FIGURE 6:**
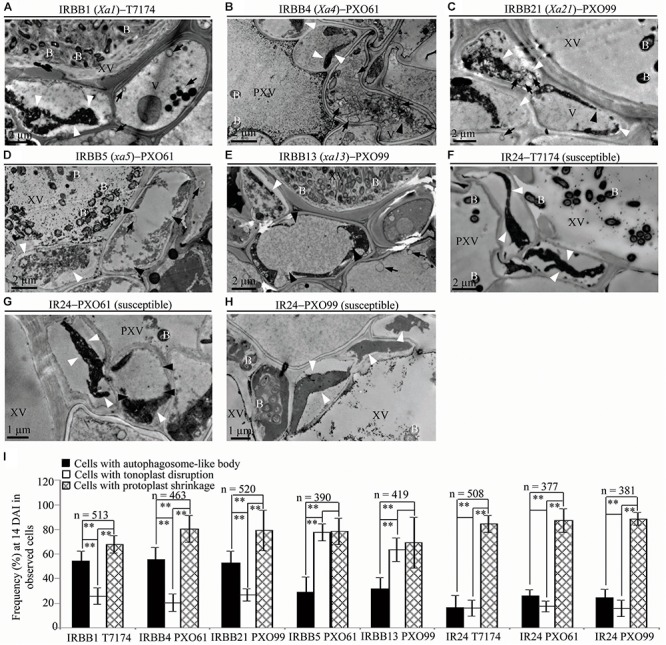
Ultrastructural features of xylem parenchyma cells in dominant *MR* genes *Xa1, Xa4*, and *Xa21* and recessive *MR* genes *xa5* and *xa13* mediated-resistance compared with the susceptible control at 14 DAI with *Xoo*. B, *Xoo* bacterium; PXV, protoxylem vessel; V, vacuole; XV, xylem vessel; dark arrow, autophagosome-like body; dark arrowhead, tonoplast disruption; and white arrowhead, protoplast shrinkage. **(A–C)** Xylem parenchyma cells with most autophagosome-like bodies and with most protoplast shrinkage in IRBB1, IRBB4, IRBB21 plants. **(D,E)** Xylem parenchyma cells with most tonoplast disruption and with most protoplast shrinkage in IRBB5 and IRBB13 plants. **(F–H)** Xylem parenchyma cells with most protoplast shrinkage in IR24 plants. (**I**) Percentage of cells with autophagosome-like bodies, tonoplast disruption, and protoplast shrinkage in micrographs of xylem parenchyma cell in rice leaves at 14 DAI with *Xoo*. Data represent mean (at least nine leaf xylem parenchyma cells were observed from nine different plants in two or three independent inoculations) ± SD. The double asterisk (^∗∗^) stands for a significant difference between frequency of cells with protoplast shrinkage and frequency of cells with autophagosome-like body or tonoplast disruption, between frequency of cells with autophagosome-like body and frequency of cells with tonoplast disruption in resistant plants, at *P* < 0.01. *n*, the total number of observed cells.

To study the ultrastructure of xylem parenchyma cells at the late stage of the rice–*Xoo* interaction and the ultrastucture of xylem parenchyma cells of rice lines at mock treatment (clipping leaf only with water), we examined the three abnormal structures, autophagosome-like bodies, tonoplast disruption, and protoplast shrinkage, in the infection sites of the rice lines at 14 DAI and the mock inoculation sites at 3, 5, 14 DAI (Figure 6 and Supplementary Figure S5). Protoplast shrinkage was observed to be the major ultrastructural feature in xylem parenchyma cells at 14 DAI of all the rice–*Xoo* interactions and all the mock treatments (Figures 6A–H and Supplementary Figures S5A–F). The number of rice cells containing protoplast shrinkage was 3- to 6-fold higher than the number of cells with autophagosome-like bodies or tonoplast disruption, respectively, in all rice lines (Figure 6I and Supplementary Figures S5G,H). However, the number of rice cells containing autophagosome-like bodies was still 2- to 3-fold higher than the number of cells containing tonoplast disruption in dominant genes *Xa1*-, *Xa4*-, and *Xa21*-mediated resistance (Figure 6I). The number of rice cells containing tonoplast disruption was still 2- and 3-fold higher than the number of cells containing autophagosome-like bodies in recessive genes *xa5*- and *xa13*-mediated resistance (Figure 6I). Furthermore, at 3 and 5 DAI, there were integrated protoplasts and intact organelles in xylem parenchyma cells and no significant difference among the numbers of xylem parenchyma cell with the three abnormal ultrastructures at mock treatment (Supplementary Figure S5).

### Effects of Autophagy Inhibitor 3-Methyladenine and Na_2_HPO_4_ Alkaline Solution on Different *MR* Gene-Mediated Resistances to *Xoo*

**FIGURE 7:**
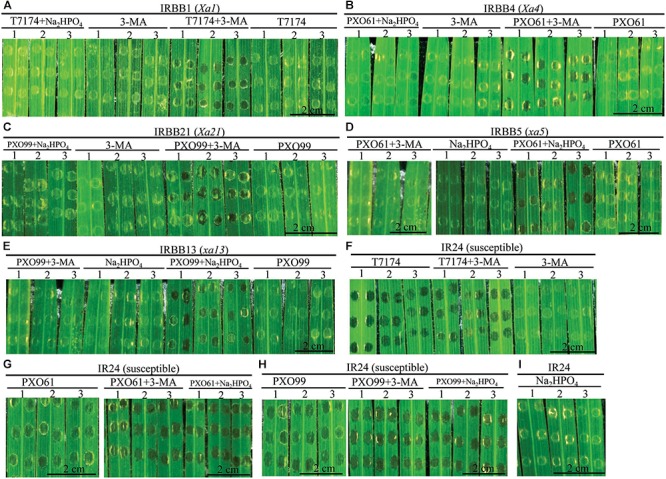
Effects of 3-methyladenine and Na_2_HPO_4_ on water-soaked symptoms in rice lines infiltrated with *Xoo* strains in different solutions at the 3rd day. **(A–I)** The symptom of IRBB1, IRBB4, IRBB21, IRBB5, IRBB13, and IR24 leaf infiltration sites with *Xoo* strains T7174, PXO61, and PXO99 (nephelometry) in H_2_O solution (T7174/PXO61/PXO99), in 5 mM 3-methyladenine (3-MA) solution (T7174/PXO61/PXO99 + 3-MA), in 2 mM Na_2_HPO_4_ solution (T7174/PXO61/PXO99 + Na_2_HPO_4_) and with only 5 mM 3-MA or 2 mM Na_2_HPO_4_ solution.

The infiltrated inoculation sites on rice leaves with deep ink-colored water-soaked symptoms defined the rice susceptibility to *Xoo* (Sugio et al., 2005; Yang et al., 2005). Na_2_HPO_4_ alkaline solution could neutralize the low pH (5.2–6.0) liquid released from disrupted vacuole (Martiniere et al., 2013). To determine if the autophagy inhibitor 3-methyladenine (3-MA) and Na_2_HPO_4_ alkaline solution could influence the resistance of rice lines with different *MR* genes, we observed whether there were water-soaked symptoms on the infiltrated inoculation sites (susceptible reaction) when the rice lines with *MR* genes were inoculated with *Xoo* bacteria in 3-MA solution or Na_2_HPO_4_ alkaline solution at 3 DAI (Figures 7A–E). The infiltration sites appeared water-soaked (deep inky color) in IRBB1, IRBB4, and IRBB21 inoculated with *Xoo* in 3-MA solution and IRBB5 and IRBB13 inoculated with *Xoo* in Na_2_HPO_4_ alkaline solution (Figures 7A–E). However, all the corresponding IRBB lines with *MR* genes inoculated with only the incompatible *Xoo* strain bacteria (resistant reaction) did not have water-soaked symptoms (Figures 7A–E). Furthermore, all the numbers of infiltration site with water-soaked symptom (susceptible reaction) in the inoculation of IRBB1, IRBB4 and IRBB21 with *Xoo* in 3-MA solution and the inoculation of IRBB5 and IRBB13 with *Xoo* in Na_2_HPO_4_ alkaline solution were significantly (*P* < 0.01) more than that in each rice lines inoculated only with *Xoo* (Supplementary Table S2). In contrast, the susceptible line IR24, when inoculated with *Xoo, Xoo* in 3-MA solution and *Xoo* in Na_2_HPO_4_ alkaline solution, had water-soaked symptoms on all infiltration sites (Figures 7F–H). Whereas there was no water-soaked symptom on infiltration sites in the rice lines only injected with 3-MA solution or Na_2_HPO_4_ alkaline solution (Figure 7).

To investigate whether the 3-MA and Na_2_HPO_4_ alkaline solution affected the ultrastructure of mesophyll cell in rice–*Xoo* interaction, we analyzed the mesophyll cell with the three abnormal structures in rice lines with *MR* genes inoculated with *Xoo* bacteria in 3-MA solution or Na_2_HPO_4_ alkaline solution at 3 DAI (Supplementary Figure S6). In rice lines with *MR* genes infiltrated by only 3-MA or Na_2_HPO_4_ alkaline solution, the mesophyll cells represented intact protoplast and the numbers of mesophyll cell with three abnormal ultrastructures did not have difference (Supplementary Figures S6A–E,I–J). However, in IRBB1, IRBB4, IRBB21 lines inoculated with *Xoo* strains, the auphagosome-like bodies were the major ultrastructural features in mesophyll cells and the numbers of mesophyll cells with autophagosome-like bodies were 3- and 4-fold higher than the number of cells containing tonoplast disruption or protoplast shrinkage (Supplementary Figures S6A–C,I). In the IRBB1, IRBB4, and IRBB21 lines inoculated with *Xoo* strains in 3-MA solution, the IRBB5, IRBB13 lines inoculated with *Xoo* strains in Na_2_HPO_4_ alkaline solution and the IR24 lines only inoculated with *Xoo* strains, there were significantly more mesophyll cells with protoplast shrinkage than the mesophyll cells with autophagosome-like body or tonoplast disruption (Supplementary Figure S6). More mesophyll cells with tonoplast disruption were observed in IRBB5 and IRBB13 lines inoculated with only *Xoo* strains, even in IRBB5 and IRBB13 lines inoculated with *Xoo* strains in Na_2_HPO_4_ alkaline solution (Supplementary Figures S6D–E,J).

These results indicated that the mixing *Xoo* bacteria in 3-MA and Na_2_HPO_4_ alkaline solution significantly reduced the dominant genes *Xa1*-, *Xa4*-, and *Xa21*- mediated resistance and the recessive genes *xa5*- and *xa13*- mediated resistance, respectively.

## Discussion

A wide variety of pathogens can lead to lesion formation on infected plant tissue and trigger hypersensitive response-programmed cell death (HR-PCD) during plant resistance against pathogens (Mur et al., 2008; Kabbage et al., 2017). Most research on HR-PCD focused on the pathogens which grow and spread in the intercellular spaces of plant cells (Kabbage et al., 2017). However, the *Xoo* bacteria multiply and spread in the xylem vessels, a vascular structure surrounded by xylem parenchyma cells in rice leaves (Niño-Liu et al., 2006; Kou and Wang, 2010). In comparison with the chlorotic water-soaked symptoms of inoculated sites of susceptible rice line, the brown HR-like lesions on the *Xoo*-infected leaves of resistant rice lines (Figure 1) indicate that *Xoo* triggered the HR-PCD of resistant rice leaf cells. The xylem parenchyma cells, which directly interact with the *Xoo* bacterium (Niño-Liu et al., 2006), and the mesophyll cells appeared to have many classic autophagosome-like bodies and tonoplast rupture structures in resistant rice lines–*Xoo* interactions (Figures 2–6 and Supplementary Figures S2, S6). *OsATG5* and *OsATG7* were only markedly induced expression in *Xa1*-, *Xa4*-, and *Xa21*-mediated resistance, as did *OsVPE2* only in *xa13*-mediated resistances (Supplementary Figure S4). Autophagy inhibitor 3-MA partially impaired the *Xa1*-, *Xa4*-, and *Xa21*-mediated resistances through reducing the number of mesophyll cells with autophagosome-like bodies (Figure 7 and Supplementary Figure S6). Meanwhile, in all the susceptible reactions, the xylem parenchyma cells showed protoplast shrinkage and plasma membrane disruption (Figures 3–6). In all the resistant rice lines, there were little cells with autophagosome-like bodies and tonoplast rupture structures in control treatment (0 DAI) and mock treatment (3, 5, 14 DAI) (Figures 3–5 and Supplementary Figures S1, S5), Therefore, the HR-PCD of xylem parenchyma cells belongs to autophagy-like cell death in *Xa1*-, *Xa4*-, and *Xa21*-mediated resistance and vacuolar-mediated cell death in *xa5*- and *xa13*-mediated resistance. However, there were approximately 70% of xylem parenchyma cells that had autophagy-like cell death and vacuolar-mediated cell death at 5 DAI (Figures 3, 4 and Supplementary Figure S2). Thus, these findings suggest that autophagy-like cell death and vacuolar-mediated cell death are the major types partly mixed with other types in resistances against *Xoo*.

The dominant *Xa1, Xa4*, and *Xa21* encode a NB-LRR-type protein, a cell wall-associated kinase and a plasma membrane-localized LRR receptor kinase, respectively (Yoshimura et al., 1998; Chen et al., 2010; Hu et al., 2017). The *Arabidopsis thaliana MR* genes *Cf9, Pto, PRS2*, and *RPS4* encode a membrane-anchored glycoprotein, a cytoplasmic serine-threonine protein kinase, a CC-NB-LRR-type protein and a TIR-NB-LRR-type protein, respectively (Pedley and Martin, 2003; Hofius et al., 2009; Chakrabarti et al., 2016). The Cf9, Pto, PRS2 and RPS4 proteins as receptors can trigger autophagy cell death to mediate *A. thaliana* resistance to *Pseudomonas syringae pathovar* (pv) *tomato, Pst* (Liu et al., 2005; Hofius et al., 2009; Gururani et al., 2012). Although XA1, XA4, and XA21 belong to different types of receptor proteins, they can accept and transfer resistance signals into rice cells during the resistance response (Yoshimura et al., 1998; Wang et al., 2006; Hu et al., 2017). As receptors, XA1, XA4, and XA21 proteins presumably have triggered autophagy-like cell death to partially mediate rice resistance against *Xoo*. The xylem parenchyma cells with autophagy-like cell death still had intact morphologies in *Xa1*-, *Xa4*-, and *Xa21*-mediated resistance (Figure 3 and Supplementary Figure S2). The intact xylem parenchyma cells possibly provide a vital cell environment to facilitate rice resistance against *Xoo*.

The low expression level of susceptible genes such as *SWEET11*/*Xa13, SWEET13*, and *SWEET14*/*Xa41* limits the growth of *Xoo* bacteria in *xa5*-, *xa13*-, and *xa41*- mediated resistances (Chen et al., 2010; Hutin et al., 2015; Huang et al., 2016; Yuan et al., 2016). The above *SWEET* genes encode glucose transporters which localize on plasma membrane and take part in pumping glucose to extracellular space (Chen et al., 2010; Hutin et al., 2015; Huang et al., 2016). A lot of other glucose transporters on tonoplast play important roles in uptake glucose into vacuole in *A. thaliana* and rice plant cells (Cho et al., 2010; Hedrich et al., 2015). The *SWEET* genes such as wheat leaf rust *R* genes *Lr67* and *Lr34* can lead to the intracellular glucose accumulation and the leaf senescence in resistance reactions (Krattinger et al., 2009; Chen et al., 2010; Moore et al., 2015). Therefore, in *xa5*- and *xa13*-mediated resistances, glucose may accumulate in xylem parenchyma cell where it is pumped into vacuole. Consequently, the tonoplast is disrupted by the high concentration of glucose in vacuole. The vacuole of plants has a low pH (5.2–6.0) to maintain the activity of acid hydrolytic enzymes and defense proteins (Neuhaus et al., 1991; Martiniere et al., 2013). These hydrolytic enzymes can inhibit *Pst* bacteria growth after being released into the extracellular matrix by tonoplast fusion with the plasma membrane in *A. thaliana* (Hatsugai et al., 2009). Meanwhile, the destructive vacuolar-mediated cell death mediated by VPE, which is characterized by vacuole collapse by tonoplast disruption and vacuole collapse leading to cytoplasmic content degradation and rapid cell death, is involved in *N. benthamiana* resistance to tobacco mosaic virus and *A. thaliana* resistance to *Pst* or *Botrytis cinerea* (Hatsugai et al., 2004; Rojo et al., 2004). We also found the tonoplast disintegration of most xylem parenchyma cells in *xa5*- and *xa13*-mediated resistances and the higher expression level of *OsVPE2* in *xa13*-mediated resistance (Figures 4, 6 and Supplementary Figure S4). Based on our results, vacuolar-mediated cell death is a form of destructive cell death. The Na_2_HPO_4_ alkaline solution (pH 9.0) dramatically increased the susceptibility but did not change the number of mesophyll cells with tonoplast disruption in *xa5*- and *xa13*-mediated resistances (Figure 7, Supplementary Figure S6 and Supplementary Table S2), which suggests that the alkaline solution partially impairs *xa5*- and *xa13*-mediated resistance against *Xoo*. Destructive vacuolar-mediated cell death may depend on the low pH created by vacuole collapse to inhibit *Xoo* growth. Alternatively, hydrolytic enzymes and defense proteins released into the cytoplasm possibly participate in *xa5*- and *xa13*-mediated resistance.

The plant cells with shrinkage of protoplast and rupture of plasma membrane is regarded as necrosis cell death (van Doorn et al., 2011). The necrosis cell death typically happens under abiotic stress (van Doorn et al., 2011; Gao et al., 2015). However, the protoplast shrinkage also occurs in fungal toxin victorin-induced cell death (van Doorn et al., 2011). Meanwhile, the cell wall deformation, protoplast shrinkage and swelling of chloroplasts are observed in susceptible reaction of a black-rot-susceptible cultivar (Golden Acre) inoculated with *Xanthomonas campestris pv.campestri* (Bretschneider et al., 1989). At 3 and 5 DAI, the xylem parenchyma cells appeared protoplast shrinkage and plasma membrane rupture only in rice susceptible line IR24–*Xoo* interactions and the rice lines with *MR* genes but susceptible to compatible *Xoo* strains (Figures 3–5). Therefore, the cell death of xylem parenchyma cells in rice lines susceptible to *Xoo* can be categorized into necrosis at early stage of infection. At 14 DAI of resistant reaction, susceptible reaction or mock treatment, most parenchyma cells of rice line all represented protoplast shrinkage (Figure 6 and Supplementary Figure S5), which indicates that the necrosis may be the general features of cell death in rice during late stages of pathogen infection and wound stress. In rice susceptible to *Xoo*, there are more bacteria in infection site than that in resistant reaction (Song et al., 1995; Yoshimura et al., 1998; Iyer and McCouch, 2004; Chu et al., 2006; Hu et al., 2017). The high bacterial population in xylem vessel of susceptible rice line may damage the xylem parenchyma cells to form necrosis. The necrosis of xylem parenchyma cells with plasma membrane rupture can possibly release nutrients into xylem vessels where *Xoo* bacteria multiply.

Plant cell often represents protoplast shrinkage and cell corpse at late stage of PCD (van Doorn et al., 2011). At 14 DAI, the leaves of infection site becoming yellow lesion (Figure 1B) indicated that the cell death of xylem parenchyma cells reached to late stage in resistant rice plants. Meanwhile, most xylem parenchyma cells appeared protoplast shrinkage and cell corpse in resistant rice plants at 14 DAI (Figure 6). But, most xylem parenchyma cells also represented autophagosome-like bodies and tonoplast disruption ultrastructures in resistant reactions (Figures 6A–E,I). Based on our results, the cell death progression in resistant rice lines happens as the following steps. Firstly, the xylem parenchyma cells always keep autophagy-like cell death or vacuolar-mediated cell death from 3 DAI to 14 DAI. Secondly, the long-time autophagosome-like body formation or tonoplast disruption processes possibly lead to the rupture of plasma membrane. Lastly, the protoplast shrinkage leads to cell corpse at late stage. However, the cell death progression of xylem parenchyma cells in susceptible rice line is always keeping necrosis i.e., protoplast shrinkage from 3 DAI to 14 DAI (Figures 2, 5, 6F–H).

## Author Contributions

JC designed and performed most of the experiments, analyzed the data, and drafted the manuscript. MZ performed the qRT-PCR assays. JX and XL provided biochemical analysis support and field management. MY revised part of the manuscript. SW supervised the project, interpreted the data, and revised the manuscript. All authors read and approved the manuscript.

## Conflict of Interest Statement

The authors declare that the research was conducted in the absence of any commercial or financial relationships that could be construed as a potential conflict of interest.

## Footnotes

1 http://www.microsoftstore.com

## Supplementary Material

FIGURE S1 Percentage of cells with autophagosome-like bodies, tonoplast disruption, and protoplast shrinkage in micrographs of xylem parenchyma cells in rice leaves at 0 DAI. Data represent mean (at least nine leaf xylem parenchyma cells were observed from nine different plants in two or three independent inoculations) ± SD. No difference between frequency of cells with protoplast shrinkage and frequency of cells with tonoplast disruption or autophagosome-like body at *P* < 0.05. *n*, the total number of observed cells.

FIGURE S2 Autophagosome-like body and tonoplast disruption ultrastructural features of xylem parenchyma cells in rice lines with *MR* genes resistant to another incompatible *Xoo* strains. V, vacuole; XV, xylem vessel; and B, *Xoo* bacterium. Dark arrow, autophagosome-like body; dark arrowhead, tonoplast disruption; white arrowhead, protoplast shrinkage; and white arrow, rupture of plasma membrane. **(A–C)** Many autophagosome-like bodies and tonoplast disruption in xylem parenchyma cells of IRBB4, IRBB21, and IRBB5 plants at 5 days after inoculation (DAI) with *Xoo* strains PXO112, PXO61, and PXO86 comparison with plants at 0 DAI. **(D)** The percentage of cells with autophagosome-like bodies, tonoplast disruption, and protoplast shrinkage in micrographs of xylem parenchyma cell in rice leaves at 0 and 5 DAI. Data represent mean (at least six leaf xylem parenchyma cells were observed from six different plants in two independent inoculations) ± SD. The double asterisk (^∗∗^) stands for a significant difference between frequency of cells with autophagosome-like body and frequency of cells with tonoplast disruption or protoplast shrinkage in at *P* < 0.01. *n*, the total number of observed cells.

FIGURE S3 Lesion length of rice lines with *MR* genes *Xa1, Xa4, xa5, xa13*, and *Xa21* infected by compatible *Xoo* strains at 14 DAI. Bars represent mean (10 to 15 leaves from four plants) ± standard deviation (SD).

FIGURE S4 Expression pattern of *OsATG5, OsATG7*, and *OsVPE2* in resistant (IRBB1, IRBB21, IRBB4, and IRBB13) and susceptible (IR24) rice lines–*Xoo* interactions. The expression of autophagy-related genes (*OsATG5, OsATG7*) and a vacuolar processing enzyme (*OsVPE2*) were analyzed by qRT-PCR in IRBB1/IR24 rice plants inoculated with *Xoo* strain T7174, IRBB21/IR24, and IRBB13/IR24 rice plants inoculated with *Xoo* strain PXO99, IRBB4/IR24 rice plants inoculated with *Xoo* strain PXO61. The rice plants were sampled on ck, 8 and 24 h after inoculation. Data are means (three replicates) ± standard deviations. ck, before inoculation. The letters “a” indicates statistically significant differences between ck and inoculated plants of the same rice plant at *P* < 0.01. Double astericks (^∗∗^*P* < 0.01) indicate statistically significant differences resistant rice plant and susceptible rice plant inoculated at same time.

FIGURE S5 Ultrastructural features of xylem parenchyma cell in rice lines with mock treatment. V, vacuole; XV, xylem vessel; and B, *Xoo* bacterium. White arrowhead, protoplast shrinkage; and white arrow, rupture of plasma membrane. **(A–F)** Many xylem parenchyma cells with protoplast shrinkage at 14 day after inoculation (DAI) comparison with the normal xylem parenchyma cells without three abnormal ultrastructures at 3 DAI and 5 DAI in IRBB1, IRBB4, IRBB21, IRBB5, IRBB13, and IR24 plants. **(G,H)** Percentage of cells with autophagosome-like bodies, tonoplast disruption, and protoplast shrinkage in micrographs of xylem parenchyma cell in rice leaves at 3, 5, and 14 DAI. Data represent mean (at least six leaf xylem parenchyma cells were observed from six different plants in two independent inoculations) ± SD. The double asterisk (^∗∗^) stands for a significant difference between frequency of cells with protoplast shrinkage and frequency of cells with tonoplast disruption or autophagosome-like body at *P* < 0.01. *n*, the total number of observed cells.

FIGURE S6 Effects of 3-methyladenine and Na_2_HPO_4_ on the ultrastructural features of mesophyll cell in rice lines infiltrated with *Xoo* strains in different solutions at the 3rd day. B, *Xoo* bacterium; V, vacuole; IS, intercellular space; Ch, chloroplast; N, nucleus; dark arrow, autophagosome-like body; dark arrowhead, tonoplast disruption; white arrowhead, protoplast shrinkage; and white arrow, rupture of plasma membrane. **(A–H)** the ultrastructural features of mesophyll cell in IRBB1, IRBB4, IRBB21, IRBB5, IRBB13, and IR24 leaf infiltration sites with *Xoo* strains T7174, PXO61, and PXO99 in H_2_O solution (T7174/PXO61/PXO99), in 5 mM 3-methyladenine (3-MA) solution (T7174/PXO61/PXO99 + 3-MA), in 2 mM Na_2_HPO_4_ solution (T7174/PXO61/PXO99 + Na_2_HPO_4_) and with only 5 mM 3-MA or 2 mM Na_2_HPO_4_ solution. **(I,J)** percentage of cells with autophagosome-like bodies, tonoplast disruption, and protoplast shrinkage in micrographs of xylem parenchyma cell in rice leaves at 14 DAI with *Xoo*. Data represent mean (at least six leaf mesohyll cells were observed from six different plants in two independent inoculations) ± SD. The double asterisk (^∗∗^) stands for a significant difference between frequency of cells with protoplast shrinkage and frequency of cells with autophagosome-like body or tonoplast disruption, between frequency of cells with autophagosome-like body and frequency of cells with tonoplast disruption in resistant plants, at *P* < 0.01. *n*, the total number of observed cells.

TABLE S1 PCR primers used for quantitative RT-PCR assays.

TABLE S2 Effect of 3-methyladenine and Na2HPO4 on the percentage of infiltrating inoculation site with water-soaked symptoms.
