# Supplementary figures and images for: Dominant and Recessive Major R Genes Lead to Different Types of Host Cell Death During Resistance to Xanthomonas oryzae in Rice
Source: Front Plant Sci. 2018 Nov 21;9:1711. doi: 10.3389/fpls.2018.01711 (PMC6258818; doi:10.3389/fpls.2018.01711)

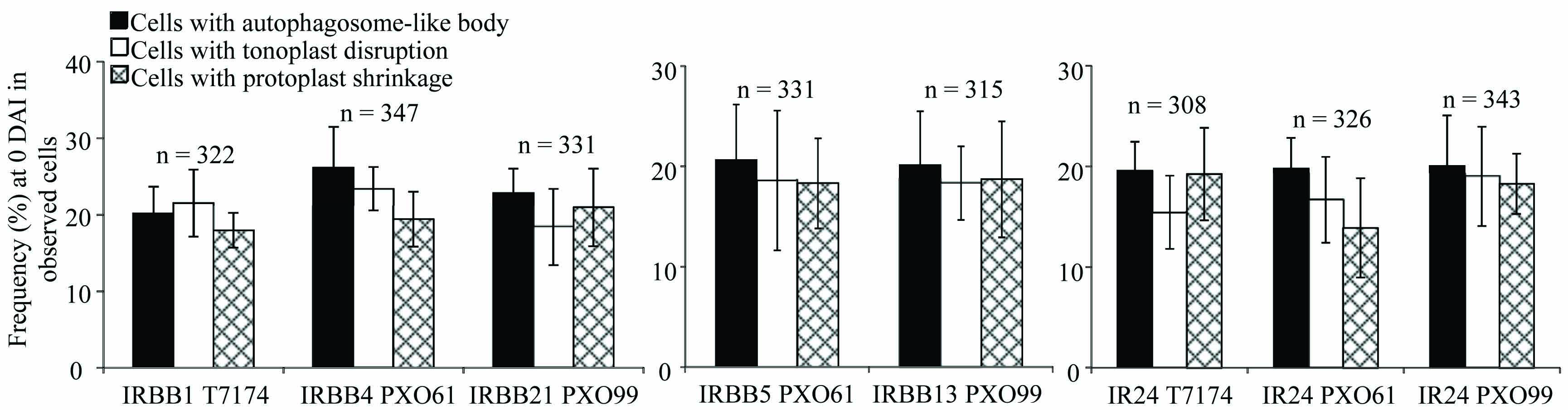

Supplement: FIGURE S1 — Percentage of cells with autophagosome-like bodies, tonoplast disruption, and protoplast shrinkage in micrographs of xylem parenchyma cells in rice leaves at 0 DAI. Data represent mean (at least nine leaf xylem parenchyma cells were observed from nine different plants in two or three independent inoculations) ± SD. No difference between frequency of cells with protoplast shrinkage and frequency of cells with tonoplast disruption or autophagosome-like body at P < 0.05. n, the total number of observed cells. [file Image_1.JPEG]

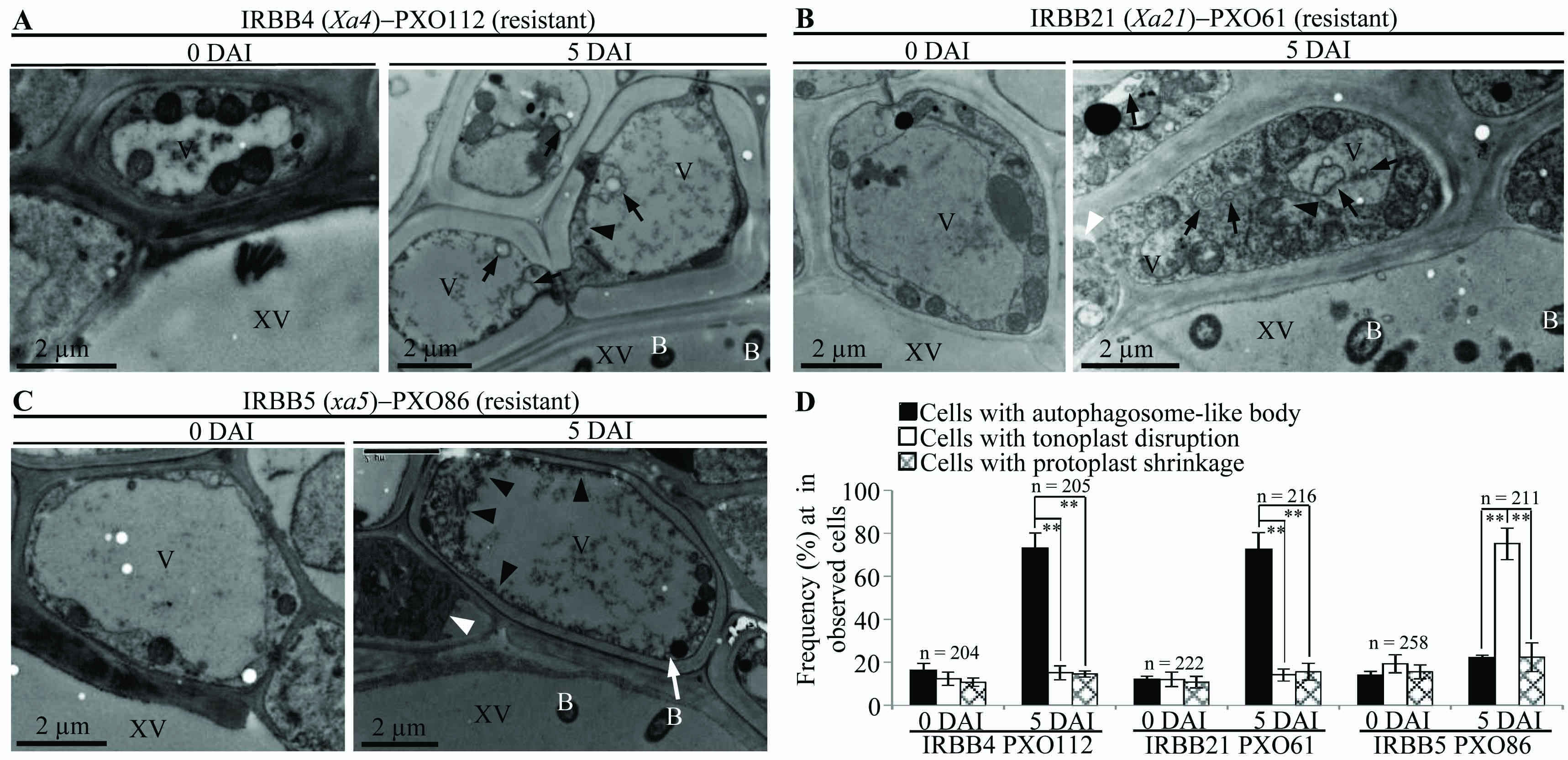

Supplement: FIGURE S2 — Autophagosome-like body and tonoplast disruption ultrastructural features of xylem parenchyma cells in rice lines with MR genes resistant to another incompatible Xoo strains. V, vacuole; XV, xylem vessel; and B, Xoo bacterium. Dark arrow, autophagosome-like body; dark arrowhead, tonoplast disruption; white arrowhead, protoplast shrinkage; and white arrow, rupture of plasma membrane. (A–C) Many autophagosome-like bodies and tonoplast disruption in xylem parenchyma cells of IRBB4, IRBB21, and IRBB5 plants at 5 days after inoculation (DAI) with Xoo strains PXO112, PXO61, and PXO86 comparison with plants at 0 DAI. (D) The percentage of cells with autophagosome-like bodies, tonoplast disruption, and protoplast shrinkage in micrographs of xylem parenchyma cell in rice leaves at 0 and 5 DAI. Data represent mean (at least six leaf xylem parenchyma cells were observed from six different plants in two independent inoculations) ± SD. The double asterisk (∗∗) stands for a significant difference between frequency of cells with autophagosome-like body and frequency of cells with tonoplast disruption or protoplast shrinkage in at P < 0.01. n, the total number of observed cells. [file Image_2.JPEG]

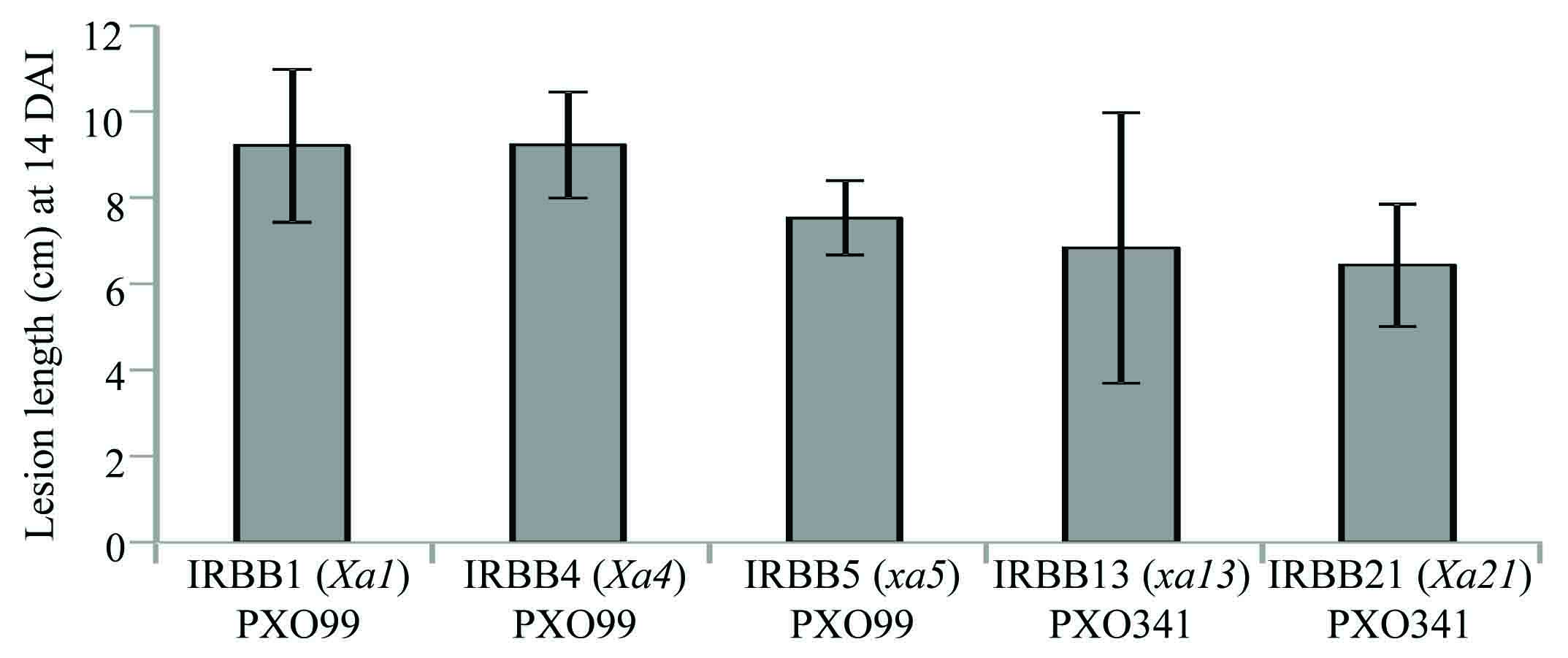

Supplement: FIGURE S3 — Lesion length of rice lines with MR genes Xa1, Xa4, xa5, xa13, and Xa21 infected by compatible Xoo strains at 14 DAI. Bars represent mean (10 to 15 leaves from four plants) ± standard deviation (SD). [file Image_3.JPEG]

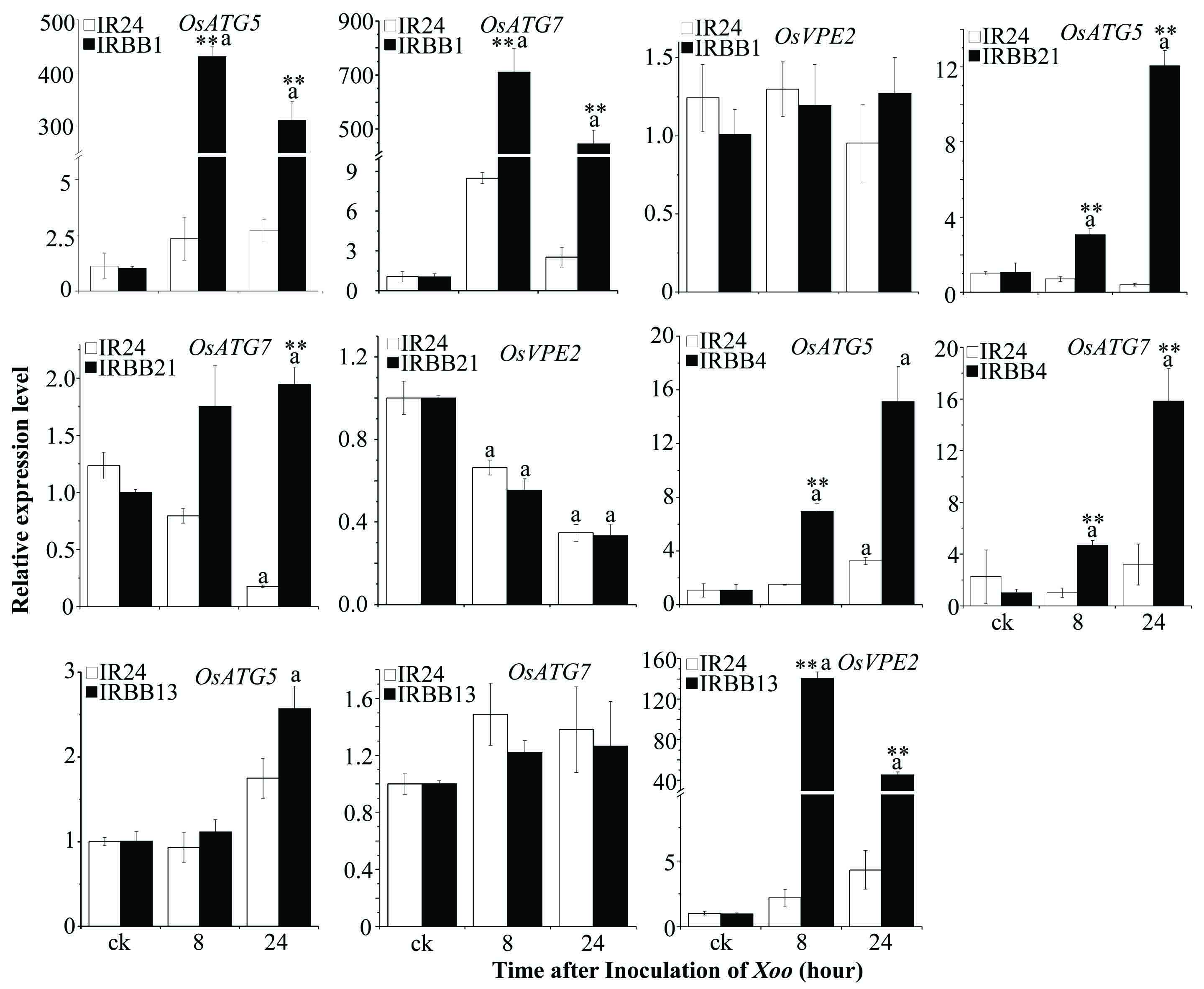

Supplement: FIGURE S4 — Expression pattern of OsATG5, OsATG7, and OsVPE2 in resistant (IRBB1, IRBB21, IRBB4, and IRBB13) and susceptible (IR24) rice lines–Xoo interactions. The expression of autophagy-related genes (OsATG5, OsATG7) and a vacuolar processing enzyme (OsVPE2) were analyzed by qRT-PCR in IRBB1/IR24 rice plants inoculated with Xoo strain T7174, IRBB21/IR24, and IRBB13/IR24 rice plants inoculated with Xoo strain PXO99, IRBB4/IR24 rice plants inoculated with Xoo strain PXO61. The rice plants were sampled on ck, 8 and 24 h after inoculation. Data are means (three replicates) ± standard deviations. ck, before inoculation. The letters “a” indicates statistically significant differences between ck and inoculated plants of the same rice plant at P < 0.01. Double astericks (∗∗P < 0.01) indicate statistically significant differences resistant rice plant and susceptible rice plant inoculated at same time. [file Image_4.JPEG]

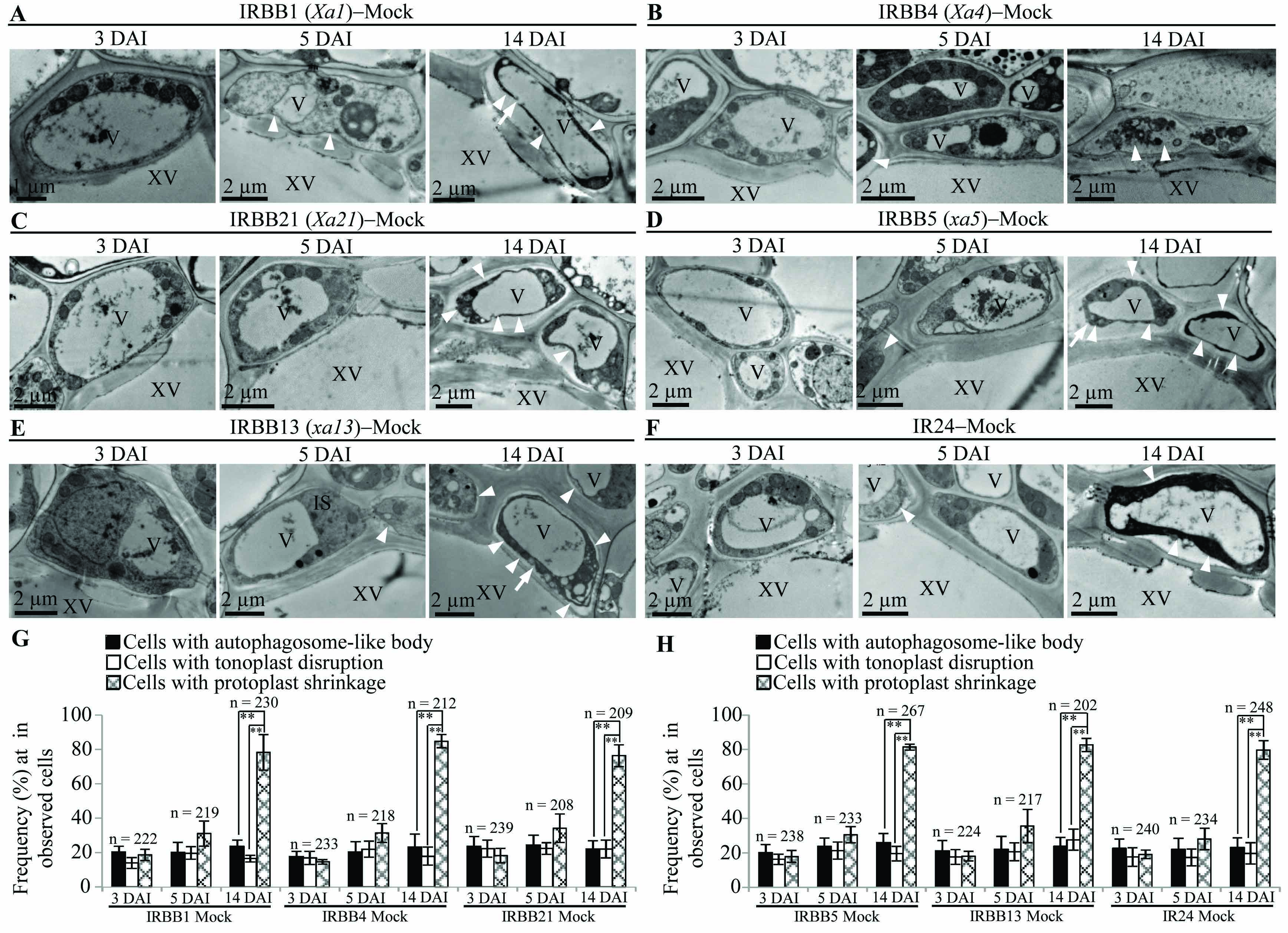

Supplement: FIGURE S5 — Ultrastructural features of xylem parenchyma cell in rice lines with mock treatment. V, vacuole; XV, xylem vessel; and B, Xoo bacterium. White arrowhead, protoplast shrinkage; and white arrow, rupture of plasma membrane. (A–F) Many xylem parenchyma cells with protoplast shrinkage at 14 day after inoculation (DAI) comparison with the normal xylem parenchyma cells without three abnormal ultrastructures at 3 DAI and 5 DAI in IRBB1, IRBB4, IRBB21, IRBB5, IRBB13, and IR24 plants. (G,H) Percentage of cells with autophagosome-like bodies, tonoplast disruption, and protoplast shrinkage in micrographs of xylem parenchyma cell in rice leaves at 3, 5, and 14 DAI. Data represent mean (at least six leaf xylem parenchyma cells were observed from six different plants in two independent inoculations) ± SD. The double asterisk (∗∗) stands for a significant difference between frequency of cells with protoplast shrinkage and frequency of cells with tonoplast disruption or autophagosome-like body at P < 0.01. n, the total number of observed cells. [file Image_5.JPEG]

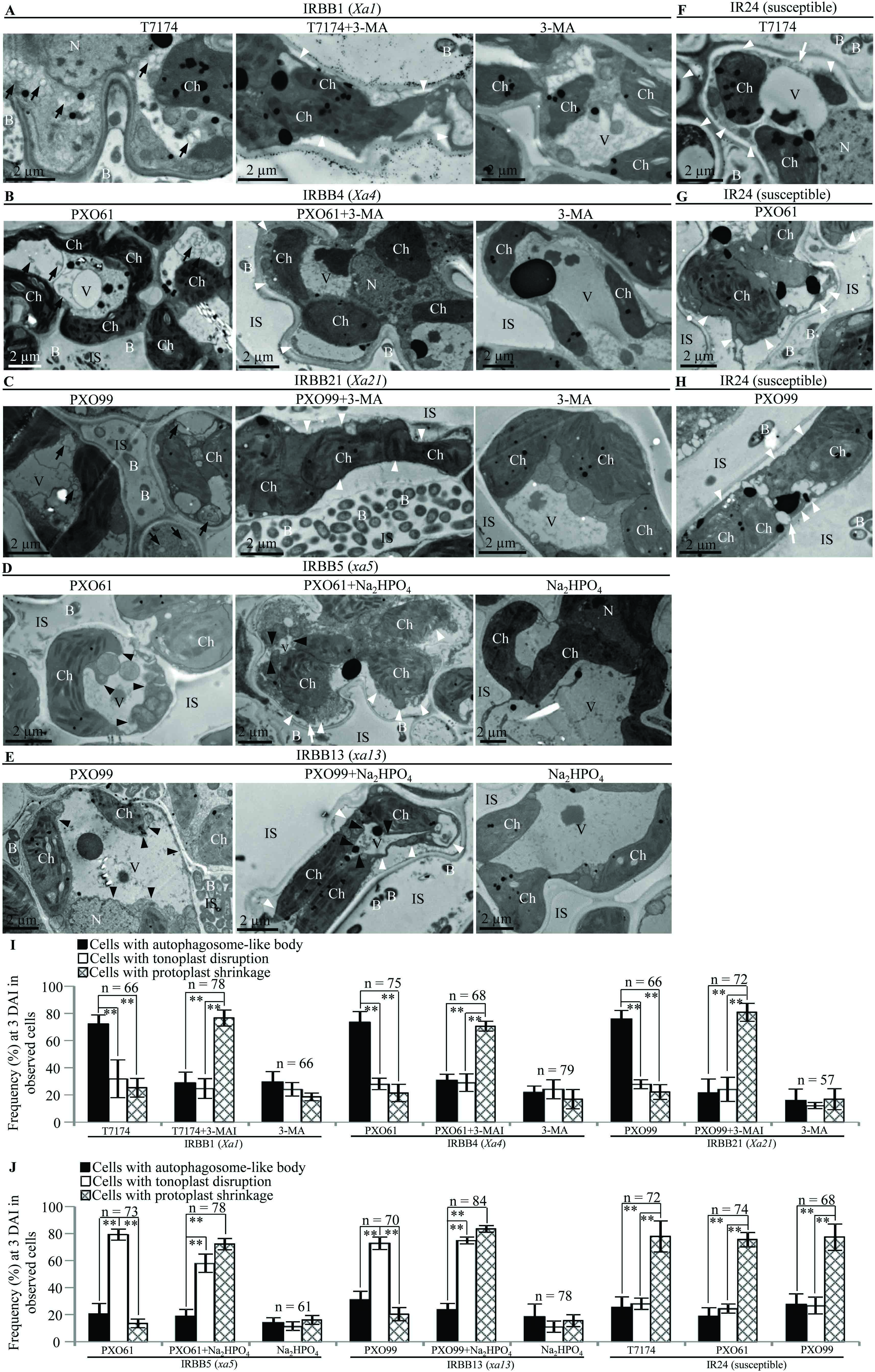

Supplement: FIGURE S6 — Effects of 3-methyladenine and Na2HPO4 on the ultrastructural features of mesophyll cell in rice lines infiltrated with Xoo strains in different solutions at the 3rd day. B, Xoo bacterium; V, vacuole; IS, intercellular space; Ch, chloroplast; N, nucleus; dark arrow, autophagosome-like body; dark arrowhead, tonoplast disruption; white arrowhead, protoplast shrinkage; and white arrow, rupture of plasma membrane. (A–H) the ultrastructural features of mesophyll cell in IRBB1, IRBB4, IRBB21, IRBB5, IRBB13, and IR24 leaf infiltration sites with Xoo strains T7174, PXO61, and PXO99 in H2O solution (T7174/PXO61/PXO99), in 5 mM 3-methyladenine (3-MA) solution (T7174/PXO61/PXO99 + 3-MA), in 2 mM Na2HPO4 solution (T7174/PXO61/PXO99 + Na2HPO4) and with only 5 mM 3-MA or 2 mM Na2HPO4 solution. (I,J) percentage of cells with autophagosome-like bodies, tonoplast disruption, and protoplast shrinkage in micrographs of xylem parenchyma cell in rice leaves at 14 DAI with Xoo. Data represent mean (at least six leaf mesohyll cells were observed from six different plants in two independent inoculations) ± SD. The double asterisk (∗∗) stands for a significant difference between frequency of cells with protoplast shrinkage and frequency of cells with autophagosome-like body or tonoplast disruption, between frequency of cells with autophagosome-like body and frequency of cells with tonoplast disruption in resistant plants, at P < 0.01. n, the total number of observed cells. [file Image_6.JPEG]
