# Supplementary material for: Dominant and Recessive Major R Genes Lead to Different Types of Host Cell Death During Resistance to Xanthomonas oryzae in Rice
Source: Front Plant Sci. 2018 Nov 21;9:1711. doi: 10.3389/fpls.2018.01711 (PMC6258818; doi:10.3389/fpls.2018.01711)
Supplement: TABLE S1 — PCR primers used for quantitative RT-PCR assays. [file Table_1.DOC]

**Supplementary Table 1.** PCR primers used for quantitative RT-PCR assays

| Gene(GeneBank accession number) | Forward primer 5’-3’ | Reverse primer 5’-3’ | Purpose |
| --- | --- | --- | --- |
| *ATG5*(AK063557) | GCCTAAGGATGGTAGATGAAATG | TCCAAGATGAGAACCAAGACC | Quantitative PCR |
| *ATG7* (AK067422) | AGTCCATCAGACGATGAGAATG | GGTAATGTTTACACTGTGATTTGC | Quantitative PCR |
| *VPE2*(AK067597) | CGGCTCCAACGGCTACTACAAC | TCGGGACCCCAGCATAGACA | Quantitative PCR |
| *Actin(X15865)* | TGRATGCCAGTGGTCGTACCA | CCAGCAAGGTCGAGACGAA | Quantitative PCR |

**Supplementary Table 2.** Effect of 3-methyladenine and Na_2_HPO_4_ on the percentage of infiltrating inoculation site with water-soaked symptoms

| Pant inoculation after 3 days**^1^** | Percentage of inoculation site with water-soaked symptom (%) ± s.d. (%)**^2^** | *P* values**^3^** | N**^4^** (inoculation sites) |
| --- | --- | --- | --- |
| IRBB1 T7174 | 16±6 | 4×10^-03^ | 54 |
| IRBB1 T7174+3-MA | 73±16 |  | 66 |
| IRBB1 3-MA | 0±0 |  | 24 |
| IRBB1 T7174+ Na_2_HPO_4_ | 19±10 |  | 36 |
| IR24 T7174 | 92±7 | 6.5×10^-01^ | 30 |
| IR24 T7174+3-MA | 94±4 |  | 30 |
| IR24 3-MA | 0±0 |  | 18 |
| IRBB4 PXO61 | 15±12 | 3.5×10^-02^ | 48 |
| IRBB4 PXO61+3-MA | 74±20 |  | 54 |
| IRBB4 3-MA | 0±0 |  | 18 |
| IRBB4 PXO61+ Na_2_HPO_4_ | 23±5 |  | 42 |
| IRBB21 PXO99 | 11±8 | 4.8×10^-04^ | 42 |
| IRBB21 PXO99+ 3-MA | 90±7 |  | 60 |
| IRBB21 3-MA | 0±0 |  | 24 |
| IRBB21 PXO61+ Na_2_HPO_4_ | 15±11 |  | 42 |
| IR24 PXO61 | 96±3 |  | 54 |
| IR24 PXO61+3-MA | 95±3 | 7.8×10^-01^ | 48 |
| IR24 PXO61+Na_2_HPO_4_ | 94±4 | 6.1×10^-01^ | 36 |
| IR24 PXO99 | 96±3 |  | 54 |
| IR24 PXO99+3-MA | 95±3 | 7.8×10^-01^ | 42 |
| IR24 PXO99+Na_2_HPO_4_ | 96±3 | 1.0×10^+00^ | 60 |
| IR24 3-MA | 0±0 |  | 30 |
| IR24 Na_2_HPO_4_ | 0±0 |  | 30 |
| IRBB5 PXO61 | 8±7 | 1.5×10^-03^ | 42 |
| IRBB5 PXO61+ Na_2_HPO_4_ | 88±10 |  | 60 |
| IRBB5 Na_2_HPO_4_ | 0±0 |  | 18 |
| IRBB5 PXO61+3-MA | 17±16 |  | 42 |
| IRBB13 PXO99 | 12±10 | 2.4×10^-03^ | 48 |
| IRBB13 PXO99+ Na_2_HPO_4_ | 79±9 |  | 72 |
| IRBB13 Na_2_HPO_4_ | 0±0 |  | 24 |
| IRBB13 PXO99+3-MA | 11±8 |  | 42 |

**1** almost 10^9^ *Xoo* strain T7174, PXO61 and PXO99 cells in water solution, in 5 mM 3-methyladenine (3-MA) solution (T7174/PXO61/PXO99+3-MA), in 2 mM Na_2_HPO_4_ solution (T7174/PXO61/PXO99+Na_2_HPO_4_) and in only 5 mM 3-MA solution (3-MA) or 2 mM Na_2_HPO_4_ solution (Na_2_HPO_4_) infiltrated into leaves of IRBB1, IRBB4, IRBB5, IRBB21, IRBB13 and IR24 plants. All plants at four-leaf stage, except for IRBB1, IRBB21 and IR24 inoculated T7174 plant at 7-leaf stage.

**2** the percentage of inoculation sites with water-soaked symptom in total infiltrated sites from at least 3 plants ± standard deviation (s.d.).

**3** *P* value for differences between plant leaves inoculated only *Xoo* strain and *Xoo* strain in 3-MA solution or Na_2_HPO_4_ solution.

**4** the number of total infiltrated inoculation sites from at least 3 independent plants.
